# Supplementary material for: Synthesis of Effect Sizes on Dose Response from Ultra-Processed Food Consumption against Various Noncommunicable Diseases
Source: Foods. 2023 Dec 12;12(24):4457. doi: 10.3390/foods12244457 (PMC10742878; doi:10.3390/foods12244457)
Supplement: Supplementary file 1 [file foods-12-04457-s001.zip › foods-2687635-supplementary.pdf]

Table S1. Inclusion Articles for Effect Size Synthesis

No	Authors	Design Study/Data Source	Population/ Years/ (Woman/ Man)	Processed Food Assesment Method	Outcomes
**Diabetes**					
1.	[16]	Prospective cohort/the French NutriNet-Sante cohort (2009-2016)	N= 104707/≥18 years/(82907/21800)	24-h food records/ NOVA food classification/weight proportion	Absolute increment 10% upf can increase risk of type 2 diabetes mellitus (HR= 1.13, 95% CI= 1.01-1.27)
2.	[17]	Prospective cohort	N= 70421/≥45 years/(41267/29154)	Food frequency questionnaire/ NOVA food classification/weight proportion	Absolute increment 10% upf can increase 25% type 2 diabetes mellitus (OR 1.17 (1.09-1.26)
3.	[18]	Prospective cohort/ Diet and Risk of Cardiovascular Diseases in Spanish (DRECE)	N= 4679//≥30 years/(2391/2288)	Food frequency questionnaire/NOVA food classification/weight proportion	10% increment (HR= 1.15, 95% CI= 1.03-1.30)
4.	[19]	Prospective cohort/UK Biobank (2007-2019)	N= 21730/40-56 years/(11299/10431)	24-h dietary recall/ NOVA food classification/weight proportion	10% increment (HR= 1.12, 95% CI= 1.04-1.20)
5.	[20]	Cross-sectional/Canadian Community Health Survey Nutrition	N= 13608/24-73 years/(6801/6807)	24-h dietary recall/ NOVA food classification/weight proportion	10% increment (OR= 1.06, 95% CI= 1.01-1.12)

			**Cancer**		
1.	[11]	Prospective cohort/French NutriNet-Sante cohort (2009-2017)	N= 104980/≥18 years (82159/22821)	24-h dietary records/ NOVA food classification/weight proportion	Absolute increment 10% upf can increase risk of overall cancer (HR= 1.12, 95% CI= 1.06-1.18)
2.	[20]	Prospective cohort/ Diet and Risk of Cardiovascular Diseases in Spanish (DRECE)	N= 4679//≥30 years/(2391/2288)	Food frequency questionnaire/NOVA food classification/weight proportion	Cancer<br>10% increment(HR= 1.19, 95% CI= 1.04-1.37)
3.	[21]	Prospective COHORT/Nurse health study (NHS)	N= 202248/30-55 years/(46341/155907)	Food frequency questionnaire/NOVA food classification/weight proportion	Colorectal Cancer<br>Men:<br>10% increment (HR= 1.04, 95% CI= 1.01-1.06)<br>Women:<br>10% increment (HR= 1.01, 95% CI= 0.98-1.04)
4.	[22]	Population-based control study	Colorectal N= 1852, BC N= 1486, PC N= 3543/55-66 years	Food frequency questionnaire/NOVA food classification/weight proportion	Absolute increment 10% upf can increase risk of colorectal cancer (OR= 1.11, 95% CI= 1.04-1.18), breast cancer (OR= 1.03, 95% CI= 0.96-1.11), prostate cancer (OR= 1.02, 95% CI= 0.93-1.12)

6.	[23]	Prospective cohort/UK Biobank	N= 15921/56-58 years	24-h dietary recall/NOVA food classification/weight proportion	**Cancer**<br>10% increment (HR= 1.02, 95% CI= 1.01-1.04)<br>**Colorectal Cancer**<br>10% increment (HR= 1.02, 95% CI= 0.97-1.06)<br>**Breast Cancer**<br>10% increment (HR= 1.00, 95% CI= 0.97-1.03)<br>**Pre-Menopausal Breast Cancer**<br>10% increment (HR= 1.00, 95% CI= 0.95-1.04)<br>**Post-Menopausal Breast Cancer**<br>10% increment (HR= 1.00, 95% CI= 0.95-1.04)<br>**Prostate Cancer**<br>10% increment (HR= 0.99, 95% CI= 0.96-1.02)
**Cardiovascular disease**					
1.	[10]	Prospective cohort	N= 105159/36-48 years/(83247/21912)	24-h dietary records/ NOVA food classification/weight proportion	Absolute increment 10% upf can increase all CVD (HR= 1.12, 95% CI= 1.05-1.20)
2.	[20]	Population-based cohort/European Prospective Investigation into Cancer and Nutrition (EPIC)/	N= 24325/64-68 years	Food frequency questionnaire/ NOVA food classification/weight proportion	10% increment (HR= 1.42, 95% CI= 1.20-1.69)

3.	[24]	Prospective cohort/Diet and Risk of Cardiovascular Diseases in Spain (DRECE)	N= 4679//≥30 years/(2391/2288)	Food frequency questionnaire/NOVA food classification/weight proportion	10% increment (HR= 1.14, 95% CI= 1.01-1.29)
4.	[25]	Prospective Cohort/Framingham offspring study	N= 3003//≥50 years	Food frequency questionnaire/NOVA food classification/weight proportion	10% increment (HR= 1.050, 95% CI= 1.020-1.080)
5.	[26]	Observational study	N= 91891/≥55-66 years	Food frequency questionnaire/ NOVA food classification/weight proportion	10% Increment (HR= 1.05, 95% CI= 1.02-1.08)
			**Obesity**		
1.	[12]	Cross-sectional/Canadian Community Health Survey	N= 19363/≥18-65 years/(8903/10460)	24-h dietary recall/klasifikasi NOVA food/weight proportion	10% Increment: obesity for high school (OR= 1.01, 95% CI= 0.96-1.07), college (OR= 1.05, 95% CI= 0.98-1.12), university diploma (OR= 1.18, 95% CI= 1.09-1.28).
2.	[18]	Prospective cohort/French Prospective Population-Based NutriNet Sante Cohort	N= 110260/≥35-48 years/(86223/24037)	24-h dietary records/ NOVA food classification/weight proportion	10% Increment: overweight (HR= 1.10, 95% CI= 1.07-1.13) dan obesity (HR= 1.10, 95% CI= 1.05-1.14)
3.	[27]	Prospective cohort study/UK Biobank	N= 22659/≥40-57 years/10844/11815)	24-h dietary recall/ NOVA food clasiification/weight proportion	10% Increment: HR= 1.10, 95% CI= 0.99-1.22

4.	[28]	Cross-sectional study/National Diet and Nutrition Survey Rolling Programme	N= 6143/≥19 years/(2961/3182)	24-h dietary recall/NOVA food clasiification/weight proportion	10% Increment: obesity (OR= 1.18, 95% CI= 1.08-1.28)
5.	[29]	Cross-sectional/Canadian Community Health Survey-Nutrition	N= 13608/<br>≥19 until ≥65 years	24-h dietary recall/ NOVA food classification/weight proportion	10% Increment: obesity (OR= 1.06, 95% CI= 1.02-1.11)
6.	[30]	Cross-sectional study/ NHANES	N= 2329/≥60 years	24-h dietary recall/NOVA food classification/weight proportion	110% increment (HR= 0.99, 95% CI= 0.99-1.01)

NOTES: BC, breast cancer; PC, prostate cancer; UPF, ultra-processed foods

Table S2. The Newcastle-Ottawa Quality Assessment Scale

1. Newcastle-Ottawa Quality Assessment Scale Cross-Sectional Studies

	Authors	Nardocci et al. 2019	Nardocci et al. 2021	Rauber et al. 2021	Hao et al. 2022
**Selection:** (Maximum 5 stars)	1. Representativeness of the case?	*	*	*	*
	2. Justice of sample size	*	*	*	*
	3. Satisfactory response rate				
	4. Validated tools for exposure assessment	**	**	*	*
**Comparability:** (Maximum 1 stars)	5. Controlling for confounding factors	*	*	*	*
**Outcome:** (Maximum 3 stars)	6. Outcome assessment	*	*	**	**
	7. Appropriate statistical test	*	*	*	*
	**Total Score**	**7**	**7**	**7**	**7**

2. Newcastle-Ottawa Quality Assessment Scale Case-Control Studies

	Authors	Romaguera et al. 2020
**Selection:** (Maximum 4 stars)	1. Is the case definition adequate?	*
	2. Representativeness of the cases	*
	3. Selection of control	
	4. Definition of control	*
**Comparability:** (Maximum 2 stars)	5. Comparability of cohorts on the basis of the design or anaysis	*
**Outcome:** (Maximum 4 stars)	6. Ascertainment of exposure	*
	7. Same method of ascertainment for cases and controls	*
	8. Non-Response rate	*
	**Total Score**	**7**

3. Newcastle-Ottawa Quality Assessment Scale Cohort Studies

	**Authors**	Fiolet et al. 2018	Beslay et al. 2020	Rauber et al. 2020	Srour et al. 2019	Srour et al. 2020	Juul et al. 2021	Levy et al. 2021	Zhong et al. 2021	Bonaccio et al. 2022	Duan et al. 2022	Ferreiro et al. 2022	Wang et al. 2022	Chang et al. 2023
**Selection:** (Maximum 4 stars)	1. Representativeness of the exposed cohort	*	*	*	*	*	*	*	*	*	*	*	*	*
	2. Selection of the non-exposed cohort	*	*	*	*	*	*	*	*	*	*	*	*	*
	3. Ascertainment of exposure	*	*	*	*	*	*	*	*	*	*	*	*	*
	4. Demonstration that the current outcome of interest was not present at start of study	*	*	*	*	*	*	*	*	*	*	*	*	*
**Comparability:** (Maximum 2 stars)	5. Comparability of cohorts on the basis of the design or analysis	*	**	**	*	*	*	*	**	*	**	*	*	*
**Outcome:** (Maximum 4 stars)	6. Assessment of outcome	*	*	*	*	*	*	*	*	*	*	*	*	*
	7. Was follow-up long enough for outcome to occur	*	*	*	*	*	*	*	*		*	*	*	*
	8. Adequacy of follow up of cohorts	*	*	*	*	*	*	*	*	*	*	*	*	*
	**Total Score**	**8**	**9**	**9**	**8**	**8**	**8**	**8**	**9**	**8**	**9**	**8**	**8**	**8**

Table S3. Food Types that Consumed

NO	AUTHORS (YEARS)	FOOD TYPES	10% INCREMENT	PARAMETERS
**DIABETES**				
1.	Srour et *al*. 2020	Unprocessed foods, culinary ingredients, processed foods, ultra-processed foods	Increment 10% consumption of upf in the total diet (kcal/d)	Body weight change dan BMI ≥ 30 kg/m2.
2.	Duan *et al*. 2022	Unprocessed foods, culinary ingredients, processed foods, ultra-processed foods (warm savory snack, traditional dutch cuisine, sweet snack, cold savory snack)	Increment of 10% consumption of upf in the total diet (kcal/d)	BMI > 26.2 kg/m2<br>Fasting glucose > 4.96 mmol/L
3.	Ferreiro et *al*. 2022	Unprocessed foods, culinary ingredients, processed foods, ultra-processed foods	Increment 10% consumption of upf in the total diet (kcal/d)	Diabetes history and reported by professional
4.	Levy et *al*. 2021	Minimally processed foods, culinary ingredients, processed foods, ultra-processed foods (bread, pastry, cake, industrial-processed chips (French fries), processed meat, pastries, cakes, dessert, biscuit, margarin and other spreads, packaged salty snacks, sauce/dressing, breakfast cereal, meat products, ready-to-eat meals)	Increment 10% consumption of upf in the total diet (kcal/d)	BMI > 27.2 kg/m2
5.	Nardocci et *al*. 2021	Unprocessed foods, culinary ingredients, processed foods, ultra-processed foods	10% increase in relative intake of ultra-processed foods (kcal/d)	BMI ≥ 30 kg/m2<br>Diabetes history and reported by a professional
CANCER				
1.	Fiolet et *al*. 2018	Unprocessed foods, culinary ingredients, processed foods, ultra-processed foods (sugary products, salty snacks, fats, processed meats, (meats fish, eggs), dairy products, ultra-processed fruits and vegetables, starchy foods and breakfast cereals, drinks)	10% increase of intake in total diet (kcal/d)	Cancer history
2.	Ferreiro et *al*. 2022	Unprocessed foods, culinary ingredients, processed foods, ultra-processed foods	Increment 10% consumption of upf in the total diet (kcal/d)	Cancer history and reported by a professional
3.	Romaguera *et al.* 2020	Unprocessed foods, culinary ingredients, processed foods, ultra-processed foods (canned vegetables, canned fish, cheese, sweet and savory snacks, ready to eat foods)	Increment 10% consumption of upf in the total diet (kcal/d)	Histology-confirmed newly-diagnoses of cancer
4.	Wang *et al.* 2022	Unprocessed foods, culinary ingredients, processed foods, ultra-processed foods (savory snacks, yogurt and dairy based desserts, ready to eat or heat mixed dishes,(meat, poultry or seafood based products), beverages, sweet snacks and desserts, (fats, condiments, and sauces)	Increment 10% consumption of upf in the total diet (kcal/d)	Report any cancer diagnosis in the previous two years from medical record.

5.	Chang *et al*. 2023	Unprocessed foods, processed culinary ingredient, processed food, ultra-processed food	Increment 10% consumption of upf in the total diet (kcal/d)	Cancer History
	**CARDIOVASCULAR DISEASE**			
1.	Srour *et al*. 2019	Unprocessed foods, culinary ingredients, processed foods, ultra-processed foods	Increment 10% consumption of upf in the total diet (kcal/d)	CVD reported
2.	Bonaccio *et al*. 2022	Unprocessed foods, culinary ingredients, processed foods, ultra-processed foods (processed meat, (cake, pies, pastries, pudding), crispbread, pizza, ice cream, dry cakes, snacks, chocolate, spreadable cheese, breakfast cereal, confectionery, sliced cheese, margarine, nut spread, biscuit, dressing)	Increment 10% consumption of pf and upf in the total diet (kcal/d)	Biomarkers in serum blood samples (cystain C, creatinine, blood glucose, total blood cholesterol, HDL-cholesterol, diastolic blood pressure, heart rate)
3.	Ferreiro *et al*. 2022	Unprocessed foods, culinary ingredients, processed foods, ultra-processed foods	Increment 10% consumption of upf in the total diet (kcal/d)	Cardiovascular disease history and reported by a professional
4.	Juul *et al*. 2021	Minimally processed foods, culinary ingredients, processed foods, ultra-processed foods	Increment 10% consumption of upf in the total diet (kcal/d)	Cardiovascular disease reported
5.	Zhong *et al*. 2021	Minimally processed foods, culinary ingredients, ultra-processed foods (cereals, soft drinks, sauces/dressings, ultra-processed dairy products, sugary products, salty snacks, margarine, meat and fish, ultra-processed fruits and vegetables, ultra-processed fruits and vegetables)	Increment 10% consumption of upf in the total diet (kcal/d)	Information on vital status by US National Death Index
**OBESITY**				
1.	Nardocci *et al*. 2019	Unprocessed foods, culinary ingredients, processed foods, ultra-processed foods (cheese, canned or preserved food, industrial packaged breads, confectionary, drinks, fast food dishes, margarine, sauces and spreads, sweetened breakfast cereal, salty snacks, reconstituted meat products, sweetened milk-based products)	10% increase in relative intake of ultra-processed food (kcal/d)	BMI ≥ 30 kg/m2
2.	Beslay *et al*. 2020	Unprocessed foods, culinary ingredients, processed foods, ultra-processed foods	Increment of 10% in the diet (kcal/d)	BMI ≥ 30 kg/m2
3.	Rauber et al. 2020	Minimaly processed foods, culinary ingredients, processed foods, ultra-processed foods (cheese, vegetables preserved in brine, processed breads, slated/smoked/canned meat and fish, ultra-processed breads, packaged pre-prepared meals, breakfast cereals, reconstituted meat products, confectionary, pastries/buns/cakes, industrial chips, drinks, milk-based drinks, salty snacks, pizza, margarine, sauce/dressing, dessert)	10% increase in the consumption (% of total energy upf)	BMI ≥ 30 kg/m2<br>WC ≥ 102 cm for men and ≥ 88 cm for women

4.	Rauber et al. 2021	Unprocessed foods, culinary ingredients, processed foods, ultra-processed (breads, snacks and desserts, frozen and shelf stable ready-to-eat/heat meals, beverages, breakfast cereals, spreads/sauces)	10% increase in the consumption (kcal/d)	BMI ≥ 30 kg/m2<br>WC ≥ 102 cm for men and ≥ 88 cm for women
5.	Nardocci e*t al.* 2021	Unprocessed foods, culinary ingredients, processed foods, ultra-processed foods	10% increase in relative intake of ultra-processed foods (kcal/d)	BMI ≥ 30 kg/m2
6.	Hao *et al*. 2022	Unprocessed foods, culinary ingredients, processed foods, ultra-processed foods	10% increase in relative intake of ultra-processed foods (kcal/d)	BMI ≥ 30 kg/m2
**NOTES:** WC, Waist Circumference; BMI, Body Mass Index				

Table S4. Ultra-Processed Foods Contribution in Weight Proportion to Nutritional Intake

ENERGY INTAKE												
NO REF	STUDY	YEAR	COUNTRY OF ORIGIN	TYPE OF STUDY	Q	UPF PROPORTION IN WEIGHT PROPORTION (%)	STUDY CODE	Hi-Mean±SD (Kcal/d)	Hi-Sample Size	Lo-Mean±SD (Kcal/d)	Lo-Sample Size	Auto
1	Fiolet et al	2018	United Kingdom	Prospective Cohort	Q2	14.3	Fiolet et al (1)	2281.1±457.7	26245	1810.6±454.1	26244	1
					Q3	19.8	Fiolet et al (2)	2480.5±472.3	26246	1810.6±454.1	26244	1
					Q4	32.3	Fiolet et al (3)	2615.8±501.8	26245	1810.6±454.1	26244	1
2	Srour et al	2019	French	Prospective Cohort	Q2	13	Srour et al (4)	1972±475.19	26418	1835.44±406.63	26396	1
					Q3	18.3	Srour et al (5)	2330.35±501.54	26326	1835.44±406.63	26396	1
					Q4	30.8	Srour et al (6)	2530.04±501.23	26019	1835.44±406.63	26396	1
3	Beslay et al	2020	French	Prospective Cohort	Q2	13.2	Beslay et al (7)	2006.5±489	27576	1816.2±475.8	27609	1
					Q3	18.7	Beslay et al (8)	2450.5±507.8	27556	1816.2±475.8	27609	1
					Q4	32.4	Beslay et al (9)	2534.7±534.5	27519	1816.2±475.8	27609	1
4	Nardocci et al[1]	2020	Canada	Cross Sectional	Q2	38.6 – 58.6	Nardocci et al (10)	2571±525.06	12923	1708.8±380.8	12923	1
					Q3	58.7	Nardocci et al (11)	2600.4±537.65	12454	1708.8±380.8	12923	1
5	Nardocci et al[2]	2020	Canada	Cross Sectional	Q2	38.6 – 58.6	Nardocci et al (12)	2682±542.06	13568	1708.8±380.8	13568	1
					Q3	58.7	Nardocci et al (13)	2900.4±565.65	13426	1708.8±380.8	13568	1
6	Levy et al	2021	United Kingdom	Prospective Cohort	Q2	15.4	Levy et al (14)	2211.1±597	5419	1799.3±565.1	5446	1
					Q3	23.6	Levy et al (15)	2570±670.5	5444	1799.3±565.1	5446	1
					Q4	41.9	Levy et al (16)	2881.6±767.6	5421	1799.3±565.1	5446	1
7	Romaguera et al	2020	Spain	Case Control	Q2	14.55	Romaguera et al (17)	2128±540	1169	1720±457	1170	1
					Q3	76.91	Romaguera et al (18)	2929±622	1204	1720±457	1170	1
8	Zhong et al	2021	America	Prospective Cohort	Q2	8	Zhong et al (19)	2116.5±555.6	18378	1440.6±646.6	18378	1
					Q3	16	Zhong et al (20)	2330±628.5	18379	1440.6±646.6	18378	1
					Q4	30	Zhong et al (21)	2680.6±724.4	18377	1440.6±646.6	18378	1
					Q5	82	Zhong et al (22)	2954±868.9	18379	1440.6±646.6	18378	1
9	Bonaccio et al	2022	Italy	Prospective Cohort	Q2	5.8	Bonaccio et al (23)	1832±482	293	1668±497	292	1
					Q3	8.6	Bonaccio et al (24)	1996±565	293	1668±497	292	1
					Q4	15	Bonaccio et al (25)	2281±548	293	1668±497	292	1
10	Duan et al	2022	Netherlands	Prospective Cohort	Q2	31.6	Duan et al (26)	2632±543	17606	1811±520	17604	1
					Q3	38.4	Duan et al (27)	2750±579	17606	1811±520	17604	1
					Q4	48.7	Duan et al (28)	2967±647	17606	1811±520	17604	1
11	Wang et al*	2022	United States	Prospective Cohort	Q2	58	Wang et al (29)	2886±604	9277	1879±569	9256	1
					Q3	93	Wang et al (30)	2985±613	9245	1879±569	9256	1

12	Wang et al**	2022	United States	Prospective Cohort	Q2	58	Wang et al (31)	2854±529	1713	528±528	13395	1
					Q3	93	Wang et al (32)	2954±524	1713	528±527	13395	1
13	Chang et al	2023	United Kingdom	Prospective Cohort	Q2	16.7	Chang et al (33)	2243.7±544.5	49356	1817.5±527	49357	1
					Q3	24.3	Chang et al (34)	2627.7±595	49357	1817.5±527	49357	1
					Q4	41.4	Chang et al (35)	2843.3±697.3	49356	1817.5±527	49357	1
**AVERAGE**								**2533.36±573.07**	**17383**	**1672.16±492.91**	**18085**	

SODIUM INTAKE

NO REF	STUDY	YEAR	COUNTRY OF ORIGIN	TYPE OF STUDY	Q	UPF PROPORTION IN WEIGHT PROPORTION (%)	STUDY CODE	Hi-Mean±SD (mg/d)	Hi-Sample Size	Lo-Mean±SD (mg/d)	Lo-Sample Size	Auto
1	Fiolet et al	2018	United Kingdom	Prospective Cohort	Q2	14.3	Fiolet et al (36)	2731.8±871	26245	2589.3±881.6	26244	1
					Q3	19.8	Fiolet et al (37)	2761.9±884.1	26246	2589.3±881.6	26244	1
					Q4	32.3	Fiolet et al (38)	2717.7±925	26245	2589.3±881.6	26244	1
2	Srour et al	2019	French	Prospective Cohort	Q2	13	Srour et al (39)	2749.9±862.6	26418	2601.1±867.6	26396	1
					Q3	18.3	Srour et al (40)	2782.7±876.9	26326	2601.1±867.6	26396	1
					Q4	30.8	Srour et al (41)	2735.3±923.7	26019	2601.1±867.6	26396	1
3	Beslay et al	2020	French	Prospective Cohort	Q2	13.2	Beslay et al (42)	2696.7±931.5	27576	2536.2±919.5	27609	1
					Q3	18.7	Beslay et al (43)	2762±962,1	27556	2536.2±919.5	27609	1
					Q4	32.4	Beslay et al (44)	2741±987,4	27519	2536.2±919.5	27609	1
8	Zhong et al	2021	America	Prospective Cohort	Q2	8	Zhong et al (45)	2404.5±919.2	18378	2171.7±934.5	18378	1
					Q3	16	Zhong et al (46)	2735.1±1058.3	18379	2171.7±934.5	18378	1
					Q4	30	Zhong et al (47)	2980.1±1237.3	18377	2171.7±934.5	18378	1
					Q5	82	Zhong et al (48)	3318.3±1457.8	18379	2171.7±934.5	18378	1
9	Bonaccio et al	2022	Italy	Prospective Cohort	Q2	5.8	Bonaccio et al (49)	2123±740	293	2062±690	292	1
					Q3	8.6	Bonaccio et al (50)	2130±760	293	2062±690	292	1
					Q4	15	Bonaccio et al (51)	2072±895	293	2062±690	292	1
13	Chang et al	2023	United Kingdom	Prospective Cohort	Q2	16.7	Chang et al (52)	1825.6±597.3	49356	151.4±769.4	49357	1
					Q3	24.3	Chang et al (53)	2027.4±639.6	49357	151.4±769.4	49357	1
					Q4	41.4	Chang et al (54)	2252.5±887.9	49356	151.4±769.4	49357	1
**AVERAGE**								**2555.13±916.67**	**24348**	**2241.41±843.77**	**24379**	

TOTAL FAT INTAKE

NO REF	STUDY	YEAR	COUNTRY OF ORIGIN	TYPE OF STUDY	Q	UPF PROPORTION IN WEIGHT PROPORTION (%)	STUDY CODE	Hi-Mean±SD (g/d)	Hi-Sample Size	Lo-Mean±SD (g/d)	Lo-Sample Size	Auto
1	Fiolet et al	2018	United Kingdom	Prospective Cohort	Q2	14.3	Fiolet et al (71)	80.3±24.4	26245	76±24.3	26244	1
					Q3	19.8	Fiolet et al (72)	82.1±25.3	26246	76±24.3	26244	1
					Q4	32.3	Fiolet et al (73)	83.4±27.3	26245	76±24.3	26244	1
2	Srour et al	2019	French	Prospective Cohort	Q2	13	Srour et al (74)	81.4±24	26418	77.2±24.1	26396	1
					Q3	18.3	Srour et al (75)	83.3±25	26326	77.2±24.1	26396	1
					Q4	30.8	Srour et al (76)	84.4±27.3	26019	77.2±24.1	26396	1
8	Zhong et al	2021	America	Prospective Cohort	Q2	8	Zhong et al (77)	53.8±24.5	18378	46±24.4	18378	1
					Q3	16	Zhong et al (78)	63.7±29.4	18379	46±24.4	18378	1
					Q4	30	Zhong et al (79)	70.3±34.9	18377	46±24.4	18378	1
					Q5	82	Zhong et al (80)	79.4±41	18379	46±24.4	18378	1
9	Bonaccio et al	2022	Italy	Prospective Cohort	Q2	5.8	Bonaccio et al (81)	75.06±1.93	293	64.85±12.14	292	1
					Q3	8.6	Bonaccio et al (82)	83.32±6.33	293	64.85±12.14	292	1
					Q4	15	Bonaccio et al (83)	84.72±7.73	293	64.85±12.14	292	1
11	Wang et al*	2022	United States	Prospective Cohort	Q2	58	Wang et al (84)	71.9±12.9	9277	66.2±15.8	9256	1
					Q3	93	Wang et al (85)	75±13.4	9245	66.2±15.8	9256	1
12	Wang et al**	2022	United States	Prospective Cohort	Q2	58	Wang et al (86)	58.1±9.4	13548	54,4±10,9	13395	1
					Q3	93	Wang et al (87)	61.1±9.9	13480	54.4±10.9	13395	1
13	Chang et al	2023	United Kingdom	Prospective Cohort	Q2	16.7	Chang et al (88)	66.4±24.3	49356	57.6±23.6	49357	1
					Q3	24.3	Chang et al (89)	71.3±26.4	49357	57.6±23.6	49357	1
					Q4	41.4	Chang et al (90)	75±30.2	49356	57.6±23.6	49357	1
AVERAGE								**74.20±23.87**	**21276**	**62.61±20.17**	**21284**	

SATURATED FAT INTAKE

NO REF	STUDY	YEAR	COUNTRY OF ORIGIN	TYPE OF STUDY	Q	UPF PROPORTION IN WEIGHT PROPORTION (%)	STUDY CODE	Hi-Mean±SD (g/d)	Hi-Sample Size	Lo-Mean±SD (g/d)	Lo-Sample Size	Auto
3	Beslay et al	2020	French	Prospective Cohort	Q2	13.2	Beslay et al (91)	32.7±12.7	27576	29.7±12.2	27609	1
					Q3	18.7	Beslay et al (92)	33.9±13.2	27556	29.7±12.2	27609	1
					Q4	32.4	Beslay et al (93)	34.74±13.9	27519	29.7±12.2	27609	1
8	Zhong et al	2021	America	Prospective Cohort	Q2	8	Zhong et al (94)	17±8.4	18378	14±8.1	18378	1
					Q3	16	Zhong et al (95)	20.4±10.2	18379	14±8.1	18378	1
					Q4	30	Zhong et al (96)	22.7±12.3	18377	14±8.1	18378	1
					Q2	82	Zhong et al (97)	25.9±14.4	18379	14±8.1	18378	1
9	Bonaccio et al	2022	Italy	Prospective Cohort	Q2	5.8	Bonaccio et al (98)	23±7	293	21±6	292	1
					Q3	8.6	Bonaccio et al (99)	25±8	293	21±6	292	1

					Q4	15	Bonaccio et al (100)	26±10	293	21±6	292	1
**AVERAGE**								**58.69±11.01**	**15704**	**20.81±8.70**	**15722**	
**FIBER INTAKE**												
**NO REF**	**STUDY**	**YEAR**	**COUNTRY OF ORIGIN**	**TYPE OF STUDY**	**Q**	**UPF PROPORTION IN WEIGHT PROPORTION (%)**	**STUDY CODE**	**Hi-Mean±SD (g/d)**	**Hi-Sample Size**	**Lo-Mean±SD (g/d)**	**Lo-Sample Size**	**Auto**
2	Srour et al	2019	French	Prospective Cohort	Q2	13	Srour et al (101)	20.1±6.9	26418	21±.7.7	26396	1
					Q3	18.3	Srour et al (102)	19.3±6.8	26326	21±7.7	26396	1
					Q4	30.8	Srour et al (103)	17.4±6.9	26019	21±7.7	26396	1
3	Beslay et al	2020	French	Prospective Cohort	Q2	13.2	Beslay et al (104)	20±7.4	27576	21±8.1	27609	1
					Q3	18.7	Beslay et al (105)	19.7±7.3	27556	21±8.1	27609	1
					Q4	32.4	Beslay et al (106)	17.7±7.4	27519	21±8.1	27609	1
7	Romaguera et al	2021	Spain	Case Control	Q2	14.55	Romaguera et al (107)	12±3.68	1169	13.6±4.18	1170	1
					Q3	76.91	Romaguera et al (108)	10.8±3.66	1204	13.6±4.18	1170	1
8	Zhong et al	2021	America	Prospective Cohort	Q2	8	Zhong et al (109)	16.6±7.6	18378	17.8±9.4	18378	1
					Q3	16	Zhong et al (110)	17.7±7.8	18379	17.8±9.4	18378	1
					Q4	30	Zhong et al (111)	18.6±8.2	18377	17.8±9.4	18378	1
					Q5	82	Zhong et al (112)	19.3±8.9	18379	17.8±9.4	18378	1
9	Bonaccio et al	2022	Italy	Prospective Cohort	Q2	5.8	Bonaccio et al (113)	20±7	293	21±7	292	1
					Q3	8.6	Bonaccio et al (114)	20±7	293	21±7	292	1
					Q4	15	Bonaccio et al (115)	19±7	293	21±7	292	1
11	Wang et al*	2022	United States	Prospective Cohort	Q2	58	Wang et al (116)	20.7±6.3	9277	23.4±8.3	9256	1
					Q3	93	Wang et al (117)	19.4±6.6	9245	23.4±8.3	9256	1
12	Wang et al**	2022	United States	Prospective Cohort	Q2	58	Wang et al (118)	17.6±5	13548	19.5±6.2	13395	1
					Q3	93	Wang et al (119)	15.9±4.9	13480	19.5±6.2	13395	1
13	Chang et al	2023	United Kingdom	Prospective Cohort	Q2	16.7	Chang et al (120)	25±10	49356	23.2±9.9	49357	1
					Q3	24.3	Chang et al (121)	26.1±10.7	49357	23.2±9.9	49357	1
					Q4	41.4	Chang et al (122)	26.1±11.8	49356	23.2±9.9	49357	1
**AVERAGE**								**19.05±7.22**	**19627**	**20.13±7.87**	**19642**	
**ADDED SUGAR INTAKE**												
**NO REF**	**STUDY**	**YEAR**	**COUNTRY OF ORIGIN**	**TYPE OF STUDY**	**Q**	**UPF PROPORTION IN WEIGHT PROPORTION (%)**	**STUDY CODE**	**Hi-Mean±SD (g/d)**	**Hi-Sample Size**	**Lo-Mean±SD (g/d)**	**Lo-Sample Size**	**Auto**
3	Beslay et al	2020	French	Prospective Cohort	Q2	13.2	Beslay et al (123)	90.9±32.7	27576	85.3±33.9	27609	1
					Q3	18.7	Beslay et al (124)	93.7±34	27556	85.3±33.9	27609	1
					Q4	32.4	Beslay et al (125)	99.1±39.1	27519	85.3±33.9	27609	1

8	Zhong et al	2021	America	Prospective Cohort	Q2	8	Zhong et al (126)	38.64±18.3	18378	29.28±16.67	18378	1
					Q3	16	Zhong et al (127)	49.14±23.33	18379	29.28±16.67	18378	1
					Q4	30	Zhong et al (128)	59.64±30.42	18377	29.28±16.67	18378	1
					Q5	82	Zhong et al (129)	83.58±58.75	18379	29.28±16.67	18378	1
9	Bonaccio et al	2022	Italy	Prospective Cohort	Q2	5.8	Bonaccio et al (130)	76±28	293	77±28	292	1
					Q3	8.6	Bonaccio et al (131)	82±33	293	77±28	292	1
					Q4	15	Bonaccio et al (132)	94±33	293	77±28	292	1
11	Wang et al*	2022	United States	Prospective Cohort	Q2	58	Wang et al (133)	49.4±30.7	9277	33±22.3	9256	1
					Q3	93	Wang et al (134)	56.6±39	9245	33±22.3	9256	1
12	Wang et al**	2022	United States	Prospective Cohort	Q2	58	Wang et al (135)	42.4±25.8	13548	32.1±21.4	13395	1
					Q2	93	Wang et al (136)	47±31.1	13480	32.1±21.4	13395	1
**AVERAGE**								**68.72±32.66**	**14471**	**52.44±24.27**	**14466**	
**CHOLESTEROL INTAKE**												
**NO REF**	**STUDY**	**YEAR**	**COUNTRY OF ORIGIN**	**TYPE OF STUDY**	**Q**	**UPF PROPORTION IN WEIGHT PROPORTION (%)**	**STUDY CODE**	**Hi-Mean±SD (mg/d)**	**Hi-Sample Size**	**Lo-Mean±SD (mg/d)**	**Lo-Sample Size**	**Auto**
8	Zhong et al	2021	America	Prospective Cohort	Q2	8	Zhong et al (137)	181.3±105.1	18378	154.9±108.2	18378	1
					Q3	16	Zhong et al (138)	211.8±122.3	18379	154.9±108.2	18378	1
					Q4	30	Zhong et al (139)	232.8±137.3	18377	154.9±108.2	18378	1
					Q5	82	Zhong et al (140)	263.8±162	18379	154.9±108.2	18378	1
9	Bonaccio et al	2022	Italy	Prospective Cohort	Q2	5.8	Bonaccio et al (141)	280±87	293	257±79	292	1
					Q3	8.6	Bonaccio et al (142)	287±97	293	257±79	292	1
					Q4	15	Bonaccio et al (143)	303±114	293	257±79	292	1
**AVERAGE**								**251.39±117.81**	**10627**	**198.66±96.69**	**10627**	

NOTES: [1], obesity; [2], diabetes; *, men; **, women; Q, Quartiles; Hi-Mean±SD, mean and standard deviation for high consume in Q2, Q3, Q4, Q5; Lo-Mean±SD, mean and standard deviation for low consume in Q1.
